# Comparison of Various Surface Treatment Procedures on the Roughness and Susceptibility to Staining of Provisional Prosthodontic Materials

**DOI:** 10.3390/jfb15090256

**Published:** 2024-09-03

**Authors:** Satheesh B. Haralur, Abdullah Turki Albarqi, Abdulellah Gharmallah Alamodi, Abdulmajeed Ali Alamri, Saad Awdah Aldail, Mohammed A. Al-Qarni, Saeed M. AlQahtani, Nasser M. Alqahtani

**Affiliations:** 1 Department of Prosthodontics, College of Dentistry, King Khalid University, Abha 62529, Saudi Arabia; smaalqahtani@kku.edu.sa (S.M.A.); nmalqahtani@kku.edu.sa (N.M.A.); 2 College of Dentistry, King Khalid University, Abha 62529, Saudi Arabia; albarqi08@gmail.com (A.T.A.); abdulellah7@gmail.com (A.G.A.); a.alaamri80@gmail.com (A.A.A.); saldail2221@gmail.com (S.A.A.); 3 Department of Restorative Dentistry, College of Dentistry, King Khalid University, Abha 61471, Saudi Arabia; maalqarny@kku.edu.sa

**Keywords:** provisional crown, bis acryl resin, poly-methyl methacrylate resin, surface roughness, color stability, spectrophotometer, polishing, surface glaze

## Abstract

Esthetically pleasing temporary prostheses are often necessary for extended periods in a variety of clinical scenarios. Adjustments to the occlusion or margins are commonly needed before cementing the temporary prosthesis. Therefore, it is clinically necessary to repolish the rough surface to avoid biological and esthetic issues associated with rough surfaces. The purpose of this in vitro study was to assess and compare the impact of various polishing protocols on the surface roughness and color stability of three resin materials used for provisional crowns. A total of 150 specimens were fabricated from auto-polymerizing polymethyl methacrylate, bis-acryl composite, and Methyl methacrylate-LC resin using a stainless steel mold. Each material group was divided into five groups (*n* = 10) based on the applied surface treatment: positive control group (G1): no roughening or surface treatment, Negative control group (G2): acrylic bur-roughened surface without any polishing, the different surface treatment groups of silicon carbide and aluminum oxide stone polishing (G3), diamond-coated rubber twist (G4), and Surface Glaze (G5). An optical profilometer was used to assess the surface roughness of all samples. After undergoing 6000 cycles of thermocycling followed by immersion in a coffee solution for 15 days at 37 °C, color parameters were measured using a spectrophotometer both before and after a storage period to evaluate color differences. A two-way ANOVA test with α = 0.05 significance level was carried out to determine the impacts of both the materials utilized and the polishing protocol. Among the three types of resin examined, the bisacryl group exhibited superior surface quality in positive control groups, while PMMA resin demonstrated higher polishability. The diamond-coated rubber twits resulted in lower Ra values of 0.36 (0.01) µm, 0.52 (0.11) µm, and 0.28 (0.05) µm for PMMA, BAMA, and MMLC resins, respectively. The application of photo-polymerized surface glaze led to a plaque accumulation threshold of 0.2 µm across all resin groups. The greatest mean color change occurred in the negative control group, indicating a propensity for more staining on rougher surfaces. The Bisacryl resin exhibited higher ΔE values, whereas PMMA showed better color stability. The lowest ΔE values were found when the surface glaze was applied to all of the provisional crown resins. Untreated Bisacryl resin exhibited the lowest Ra values, while PMMA resins demonstrated superior surface morphology after polishing. PMMA provisional crown resins showed increased resistance to staining. The use of surface glaze enhanced both smoothness and color stability on the surfaces.

## 1. Introduction

Interim prostheses play a crucial role in safeguarding prepared teeth and periodontal tissues, and in ensuring the maintenance of oral function and appearance [1]. Few dentists currently employ computer-aided design and manufacturing technology to produce provisional prostheses using subtractive or additive methods. However, the majority of clinicians still prefer traditional fabrication techniques that utilize different polymers due to their cost-effectiveness, availability, and ease of production [2]. The different types of resin materials used for fabricating temporary restorations include auto-polymerizing polymethyl methacrylate, polyvinyl methacrylate, polyethylene methacrylate, bis-acryl, urethane methacrylate, and microfilled resin [3]. Interim-fixed dental prostheses are frequently employed for prolonged periods in situations involving implant-supported restorations and extensive prosthetic rehabilitation [4]. Numerous clinical scenarios, such as extensive prosthetic rehabilitation, hard and soft tissue augmentation to optimize the implant site prior to surgery, and preparation of the pontic area to enhance esthetics and emergence profile, necessitate the use of long-term provisional prostheses. To function as a dependable provisional solution over the long-term, the interim restoration must possess key characteristics such as exceptional wear resistance, mechanical durability, and biocompatibility. Furthermore, it should have good esthetics to ensure overall patient satisfaction.

For interim materials utilized in the esthetic area, exceptional staining resistance is mandatory to avoid discoloration following manufacturing. Discoloration can result in suboptimal esthetics and patient discontent, necessitating the replacement of the prosthetic device [5]. The stain resistance of temporary restorative materials is determined by multiple factors and is influenced by the manufacturing process, material composition, degree of polymerization, oral hygiene practices, dietary habits, water absorption, and surface texture [6,7]. While autopolymerizing resins are a popular choice in clinical practice, they do exhibit notable shrinkage and heat production during polymerization [8]. As a result, adjustments are often necessary both prior to and following cementation. These adjustments may include contour shaping, correcting occlusal discrepancies, enhancing esthetics, and refining margins. Reports suggest that color stability is closely associated with surface roughness [9] and impacts the material’s biocompatibility. The roughness of a surface is closely related to its surface spectral reflectance, which, in turn, determines the color of an object [10]. Chung et al. [11] reported that finishing and polishing procedures during restorative treatment can affect the optical properties of dental resin composites. The rough surface not only modifies the material’s optical properties but also enhances plaque accumulation and gingival irritation [12], which can compromise the predictability and outcome of the final prosthesis. It is imperative to attain a smooth surface on restorative materials through different finishing and polishing techniques. Nevertheless, utilizing conventional abrasive-based polishing techniques may lead to the development of microcracks and small imperfections [13]. In order to enhance resistance to discoloration and elevate surface characteristics, the use of sealant agents has also been suggested [14]. Research studies have explored the effects of discoloration agents such as tea, coffee, and red wine on provisional materials [1]. Rutkunas V et al. [15] discovered that traditional temporary material bis-acryl-methacrylate-based resins exhibited better color stability than methyl/ethyl methacrylate-based resins. Conversely, Sham AS et al. [16] demonstrated that methacrylate-based resins exhibited exceptional color stability, irrespective of the staining agent or polishing approach employed. Few authors [17] have reported that materials based on bis-acryl and Bis-glycidyl methacrylate demonstrate enhanced mechanical properties and improved color stability when compared to acrylic resins. Polymer-based resin materials are the predominant constituents of provisional crowns, leading to polymerization shrinkage that can result in discrepancies in occlusal and marginal fit. This often necessitates moderate corrections to the provisional prosthesis before cementation. Additionally, surface roughness can significantly impact water sorption properties, potentially causing variations in the refractive index between the filler and matrix, and encouraging bacterial accumulation [18]. This, in turn, can affect the esthetics and hygienic potential of the provisional restoration. Consequently, it is imperative for the dentist to identify appropriate polishing protocols for each provisional crown resin material and investigate the effectiveness of these procedures on surface texture and color stability. The outcomes of the research would enable dentists to make evidence-based decisions regarding the appropriate polishing protocol for temporary crowns, resulting in a smooth surface finish that enhances color stability.

The aim of this in vitro study was to assess the surface roughness values of different conventional provisional crown materials following various surface polishing techniques and evaluate their influence on color stability in coffee solutions. The null hypotheses were that the surface roughness of interim materials would remain unaffected by the resin material type or surface treatment and that their color stainability would not be impacted by variations in the resin material, surface treatment, or storage solution.

## 2. Materials and Methods

**Figure 1:**
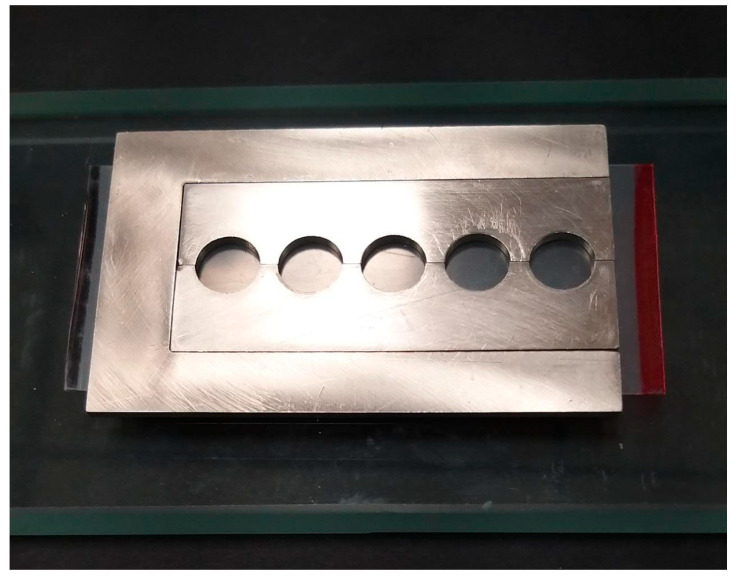
Stainless steel mold utilized to fabricate the provisional resin disc samples.

The research protocol received approval from the Institutional Review Board at the College of Dentistry, King Khalid University, under ethical review waiver IRB/KKUCOD/ETH-W/2022-23/015. A total of 50 disc-shaped specimens measuring 10 mm diameter × 2.00 mm thickness for each material were fabricated using a cylindrical stainless steel mold (Figure 1). Three commercially available provisional materials were tested: Polymethyl methacrylate [Trim^®^ Plus, Keystone Industries, Gibbstown NJ, USA], Bisacryl methacrylate (Success CD, Promedica Dental Material GmbH, Neumuenster, Germany], and Methyl methacrylate-light-cure resins [UNIFAST™ LC. GC Corporation, Bunkyo-ku, Tokyo, Japan] (Table 1).

### 2.1. Fabrication of Provisional Resin Samples

**Figure 2:**
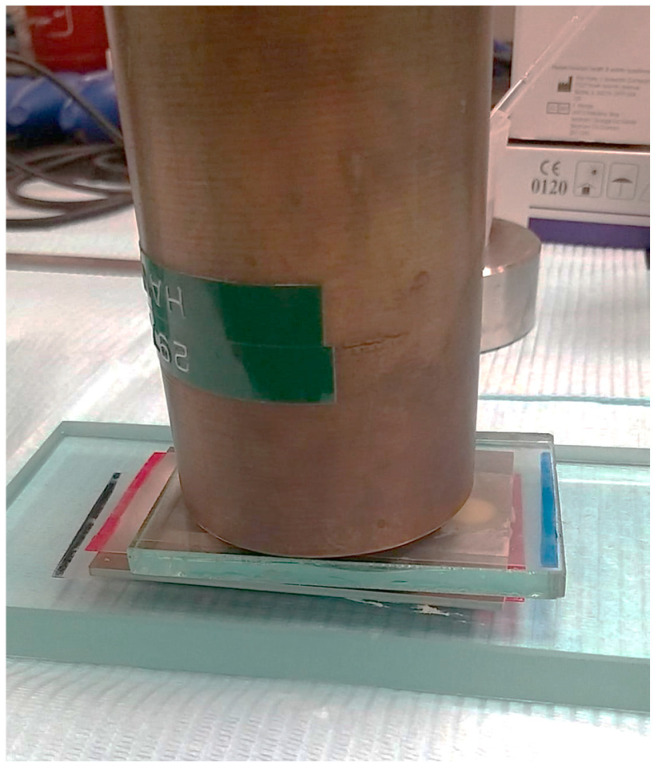
Process to fabricate the provisional resin disc samples.

Following the manufacturer’s instructions, the monomer and powder were mixed for 20 s until a uniform mixture was achieved. Polymethyl methacrylate (PMMA) resin was mixed with a powder/liquid ratio of 2.1 g to 1 mL of liquid, while Methyl methacrylate-LC(MMLC) resins used a powder/liquid ratio of 1.0 g to 0.5 mL The material was then packed into the mold once it reached the dough stage and covered with a glass plate (Figure 1). A constant load of 4 kg was applied to the glass slab to extrude excess material, flatten the surface, and reduce voids (Figure 2). PMMA specimens were extracted following a 30-min polymerization process, while the MMLC resin was cured using a visible light-curing device (Elipar S10, 3M ESPE, St. Paul, MN, USA) with a wavelength of 430–480 nm, and 1200 mW/cm^2^ light intensity for 60 s. Following polymerization, the glass slabs were detached, and air pressure was utilized to remove the samples from the mold. Bisacryl methacrylate (BAMA) resin samples were fabricated by dispensing materials directly into the metal mold using a dispensing gun. The mold, along with the material, was then covered with a glass slab, and a 4 kg weight was placed on top of the assembly. After allowing the specimen to polymerize for 5 min, the glass slabs were separated, and air pressure was used to retrieve the specimen from the mold. The specimen’s surface was cleaned with a 70% alcohol swab to eliminate the partially polymerized layer inhibited by oxygen.

**Figure 3:**
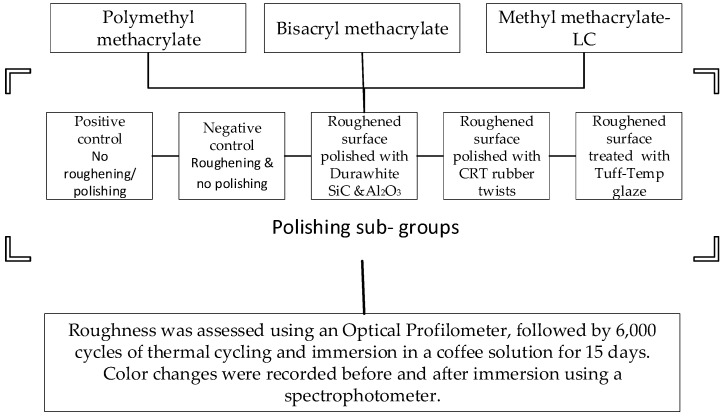
Methodology flowchart with sample distribution.

**Figure 4:**
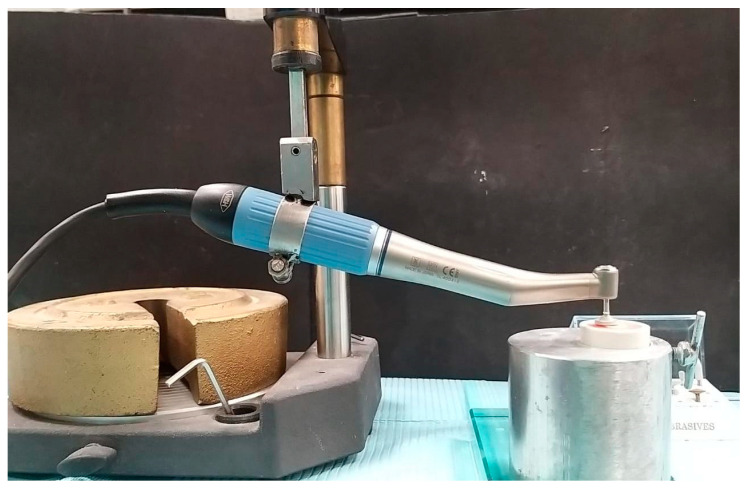
Custom-made apparatus to polish the resin disc samples.

A chlorinated PVC cylinder with self-curing poly(methyl methacrylate) acrylic resin was used to secure the resin during the polishing procedure. Once the polishing process was completed, the resin discs were carefully extracted from the cylinder for further analysis. The provisional crown samples in the positive control (G1) group (*n* = 10) were neither roughened nor subjected to any polishing procedure or glaze application (Figure 3). In contrast, the negative control (G2) group samples (*n* = 10) were roughened with an acrylic trimming bur (K79GSQ, Komet USA, Rock Hill, SC, USA) for 20 s at 20,000 RPM by a single researcher and not subjected to any polishing procedures. The control groups were used to represent the provisional crown resin surfaces, both before and after clinical modifications. The positive control group mimicked the unaltered provisional crown resin, while the negative control group simulated the surfaces following diamond point correction post-occlusal adjustments. The samples of Group-3 (G3) were first roughened and then polished using a polishing kit (DW, Durawhite, SHOFU INC., Higashiyama-ku, Kyoto, Japan) which contained silicon carbide and aluminum oxide stones. To standardize the polishing procedure, a custom-made device (Figure 4) was used. The custom-made polishing device utilized a W&H micromotor (W&H Dentalwerk Bürmoos GmbH, Bürmoos, Austria) fixed to a vertical holding device with adjustable RPM control under the constant load of 20 N. The composite and composite fine polishers were sequentially applied for 40 s each at a speed of 20,000 RPM. The Group 4 (G4) roughened samples (*n* = 10) underwent polishing using rubber polishing twists (CRT, CAREPACY, Union Sunlight Inc., South El Monte, CA, USA) with different diamond grits including coarse, medium, and fine. The smoothing, pre-polishing, and high gloss polishing twists were applied sequentially at 8000 RPM for 40 s each using a handpiece mounted on a custom-made device. The Group 5 (G5) sample was smoothed using a blend of acrylic polishing and finishing with a Carbide Cutter (SHOFU INC., Higashiyama-ku, Kyoto, Japan). This was then followed by the application of provisional glaze (Tuff-Temp glaze, PULPDENT Corporation in Watertown, MA, USA ). A thin layer of provisional glaze was applied to the samples and then dispersed using a gentle air spray. The glaze was subsequently light-cured for 20 s. The sample size of 10 for each subgroup was calculated based on previous studies [19,20]. The G* Power software (version 3.1; University of Dusseldorf) was employed to estimate the sample size using a moderate effect size (d) of 0.4, α of 0.05, and a power (1-β) of 0.73 [21]. The effect size was calculated by comparing the surface roughness measurements of the control and polished groups [19,20].

### 2.2. Surface Roughness Assessment

**Figure 5:**
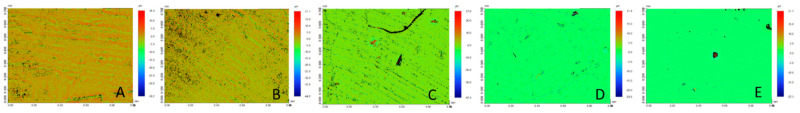
Three-dimensional optical profilometer surface topography images of (**A**)_positive control, (**B**)_negative control, (**C**)_DW, (**D**)_CRT, and (**E**)_GA polished groups from PMMA provisional resin.

**Figure 6:**
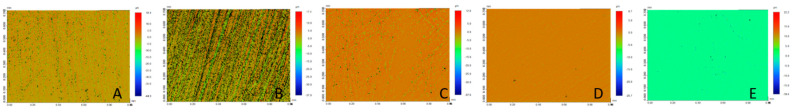
Three-dimensional optical profilometer surface topography images of (**A**)_positive control, (**B**)_negative control, (**C**)_DW, (**D**)_CRT, and (**E**)_GA polished groups from BAMA provisional resin.

**Figure 7:**
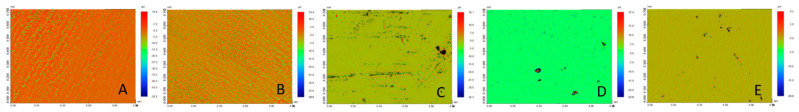
Three-dimensional optical profilometer surface topography images of (**A**)_positive control, (**B**)_negative control, (**C**)_DW, (**D**)_CRT, and (**E**)_GA polished groups from MMLC provisional resin.

After treating the temporary resin samples with surface modification based on their respective groups, the provisional crown resin specimens were first subjected to a thorough 10-min cleaning process in an ultrasonic cleaning device (W&H Dentalwerk Bürmoos GmbH, Bürmoos, Austria) utilizing deionized water. Following the cleaning, the specimens were then allowed to air-dry for a duration of 20 s to ensure the complete removal of any remaining moisture and loose debris. A Contour GT-K 3D Optical Profilometer (Bruker, Rudolf-Plank-Str. 27, Ettlingen, Germany) was utilized for the characterization and imaging of surfaces using non-contact 3D surface metrology. Vertical scan interferometry with a 5× Michelson magnification lens and a field of view measuring 1 × 1 mm, along with Gaussian regression filter, scanning at a speed of 1× and thresholding at 4, were employed to measure samples. The samples were positioned on the platform and manually adjusted to generate an image on the microscope’s display. An interferometer, utilizing a white broadband light source, was used to assess the object’s surface roughness, as well as to determine height differences between adjacent pixels exceeding 135 nm. The average roughness value for each sample was calculated by conducting three scans and averaging the results (Figure 5, Figure 6 and Figure 7).

### 2.3. Colour Measurement

The temporary resin samples were assessed for color evaluation subsequent to the measurement of surface roughness. CIELAB color attributes were assessed at two points: initial baseline color measurement and after being immersed in a coffee solution for 15 days at 37 °C. Before being placed in the staining solution, the samples underwent 6000 cycles of thermal cycling (SD Mechatronik, Feldkirchen-Westerham, Germany) in a water bath ranging between 5 and 55 °C with a dwell time of 30 s. A coffee (Nescafe Classic, Nestle Middle East manufacturing LLC, Dubai, United Arab Emirates) solution was prepared for the discoloration test by blending 15 g of powder with 200 mL of warm water. Over a period of 15 days, the solution was stirred at regular intervals (2–3 times) to ensure uniformity [22]. The CIELAB color was determined using the spectrophotometer (Lab Scan XE, Hunter Associates Laboratory, Inc. Sunset Hills Road, Reston, VA, USA). This advanced spectrophotometer operates within a light wavelength range of 400 to 700 nm and has a measurement diameter of 5 cm with reflected color measured using 0°/45° geometry. The formula was utilized to determine the color change resulting from coffee solution immersion for 15 days. ΔE = ([ΔL*]2 + [Δa*]2 + [Δb*]2) × ½ [23]. A ΔE value of 3.5 units is considered the threshold for visually perceptible color change [24].

### 2.4. Statistical Analysis

The data analysis was performed using SPSS 19 software (IBM Corp, Armonk, NY, USA). A two-way ANOVA test was conducted to determine the effects of the materials used, polishing protocol, and combined interaction. Tukey’s post-hoc examination was carried out to identify significant differences among the experimental groups. The significance level was set at *p* < 0.05.

## 3. Results

Table 2 displays the average and standard deviations of mean roughness values for various categories of provisional resin groups. The untreated positive control groups exhibited moderately lower mean surface roughness across all the resin materials. For instance, the mean Ra values for the positive control group of PMMA were recorded at 1.62 ± 0.22 µm, while BAMA and MMLC samples registered 1.41 ± 0.18 µm and 1.55 ± 0.14 µm, respectively. As anticipated, there was a significant increase in mean roughness values in the acrylic bur-roughened (negative control) group samples with corresponding Ra values for PMMA, BAMA, and MMLC groups being 2.42 ± 0.23 µm, 2.59 ± 0.20 µm, and 2.34 ± 0·19 µm, respectively. All the polishing methods used in the research led to a significant decrease in average roughness values. The use of a DW polishing kit with silicon carbide and aluminum oxide stones resulted in a moderate reduction in Ra values for all resin groups, while CRT groups using diamond-impregnated rubber twists displayed comparatively smoother surface characteristics. The mean Ra values for DW and CRT polishing techniques were 0.86 ± 0.12 µm and 0.36 ± 0.11 µm for PMMA resin, respectively, followed by 1.10 ± 0.27 µm and 0.52 ± 0.11 µm for BAMA resins. The resin group with the lowest Ra values was GA with glaze application, recording 0.17 ± 0.07 µm for PMMA, 0.15 ± 0.04 µm for BAMA, and 0.24 ± 0.04 µm for MMLC resins.

**Figure 8:**
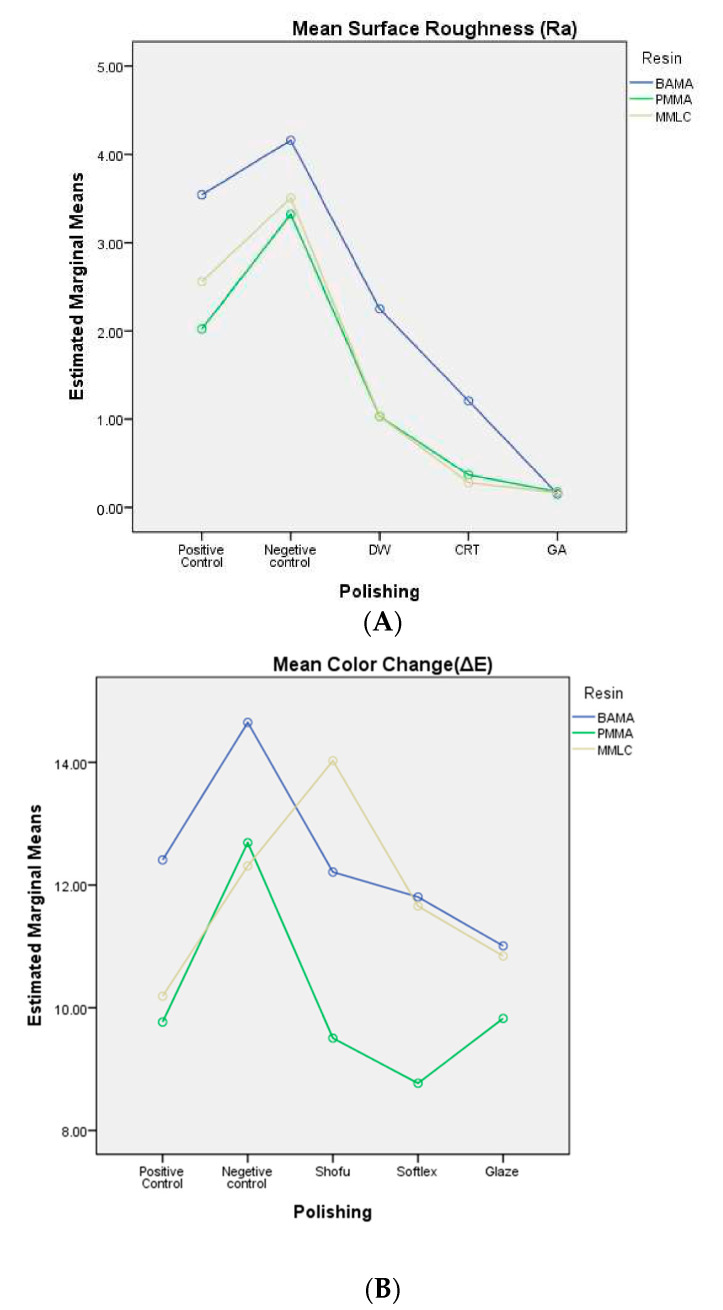
(**A**): Two-way ANOVA analysis for interaction resin and polishing over mean surface roughness (Ra); (**B**): two-way ANOVA analysis for interaction resin and polishing over mean color change (ΔE).

Table 3 presents the mean color changes(ΔE) when immersed in a coffee solution from the baseline color. The untreated negative control groups within all provisional resin groups showed higher ΔE values compared to positive control and polished groups. Across all surface treatment protocols, BAMA resin exhibited the highest ΔE value in the negative Control group (8.50 ± 0.59), followed by DW (7.01 ± 0.59), CRT (5.87 ± 0.44), and GA (5.39 ± 0.63) groups among the tested resins. Considering PMMA provisional crown resin’s performance concerning color stability, it displayed better results with the greatest ΔE of 6.59 ± 0.57 in the negative control group down to as low as 3.92 ± 0.55 in the GA group. Upon assessing different surface treatment procedures, it was noted that using Carbide disk smoothing in combination with glaze application led to the lowest ΔE values for the majority of resin categories. The Shapiro–Wilk test confirmed that the data distribution was normal at a significance level of 0.05. The two-way Anova evaluation of Ra values (Figure 8A) showed a significant main effect for the type of provisional resin (*p* = 0.001), F(2, 3.360) = 7.985, with a partial η2 value of 0.106, a significant main effect for different polishing protocols (*p* = 0.000), F(4, 3.360) = 1031.274, and high partial η2 value of 0.968, and a significant interaction between resin type and polishing protocol (*p* = 0.000), F(8, 3.360) = 4.883 with a partial η2 value of 0.224. Similar findings from a two-way ANOVA analysis (Figure 8B) on ΔE values also showed a significant impact on the type of provisional resin and the polishing protocols. The results revealed a significant interaction between resin type and polishing protocol (*p* = 0.000), F(8, 35.664) = 1.128, with a partial η2 value of 0.202. Statistical analysis using Tukey’s HSD post-hoc test (Table 4) revealed significant differences in surface roughness between the PMMA and BAMA polishing groups, while the MMLC resin group showed no significant difference compared to the PMMA and BAMA resin groups. Similarly, the Tukey’s HSD post-hoc analysis (Table 5) of color changes recorded statistically significant differences among all the resin group samples, except between the PMMA and MMLC resin groups.

## 4. Discussion

Numerous clinical scenarios necessitate the long-term provisionalization of prepared teeth. Inexpensive polymerized resin materials are often utilized for fabricating provisional crowns. However, among other drawbacks of these materials, their polymerization shrinkage can adversely affect the fit and occlusal relationships, thus routinely requiring modification of the occlusal and marginal areas of the crowns. Adequate finishing and polishing are crucial to achieving optimal surface properties, stain resistance, and preventing plaque accumulation. Researchers suggest maintaining a surface roughness of less than 0.2 µm to minimize plaque accumulation [25]. This in vitro investigation examined the impact of various repolishing protocols on the surface roughness and color stability of different provisional resin materials. The study’s findings indicated that the surface roughness and color stability of provisional resin materials were affected by both the material itself and the surface treatment protocol. The hypothesis stating that various polishing methods do not impact the surface roughness and color stability of provisional resin materials was found to be invalid.

The study revealed that BAMA resin produced a significantly smoother surface by polymerizing against a smooth matrix and then cleaning the surface with 70% alcohol to remove the oxygen inhibition layer compared to PMMA and MMLC resin samples [26]. BAMA resins containing nanofillers in their composition produced a smoother surface appearance. Other studies [27] have also found that BAMA have a notably smoother surface compared to PMMA temporary acrylic resins, possibly due to the presence of air bubbles and porosity during the hand-mixing process. BAMA resins have an advantage due to their auto-mixing system, which prevents the incorporation of air bubbles. The porosity of provisional crown resins can influence their roughness and color stability. Porosity is an undesirable consequence of the fabrication and polymerization processes, and can result from factors such as air entrapment during mixing, contraction, and vaporization of the monomer, residual monomer, and insufficient mixing or pressure. These porous surfaces create a favorable environment for bacteria to grow, which in turn affects the color stability of the provisional crown resins

Korkmaz et al. [28] proposed the use of a rough negative control group to replicate the wear caused by rotary instruments in the mouth during occlusal corrections. The roughening with an acrylic trimming bur resulted in a significant increase in Ra values across all groups, as reported in previous literature [19,29]. However, BAMA resin showed a higher increase in Ra value from 1.41 µm to 2.59 µm. The harder filler particles in comparison to the resin matrix may result in selective removal of the resin matrix during finishing and polishing, which exposes the filler on the surface. This exposure of larger filler particles can contribute to an elevation in surface roughness values. Moreover, these exposed filler particles are vulnerable to being removed from the surface during the polishing process using an acrylic bur or disc [9]. The process of polishing using silicon carbide and aluminum oxide stones effectively reduced surface roughness in all provisional resin groups tested. Notably, PMMA resins exhibited a substantial reduction in Ra values of up to 0.86 µm. Gantz et al. [27]. determined that the polishing process resulted in the removal of air bubbles and porosity in PMMA and MMLC resin, resulting in a smoother surface, as observed through AFM. This finding is consistent with that of Rutkunas et al. [15], who found that using silicone tips for polishing bis-acryl resins resulted in higher roughness values. Methacrylate resins, being unfilled, can be effectively polished using traditional techniques to achieve a smooth surface without voids [30]. A study by Haselton et al. [31] evaluated the surface roughness of methacrylate and BAMA after a two-week storage period in artificial saliva and artificial saliva-coffee solution. The results revealed that, in general, methacrylate resins exhibited smoother surfaces after initial polishing in comparable conditions. The optimal surface topography with roughness below the recommended 0.2 µm was achieved in all samples using photopolymerized surface glaze. The current investigation found that PMMA and MMA had Ra values of 0.17 µm and 0.24 µm, respectively, whereas the BAMA resins showed a Ra value of 0.15 µm. The glazed resin restoration process involves applying a light-curing, transparent resin coating that permeates the surface and fills micropores and defects [32]. This aids in reducing porosity and microleakage on the surface. The glazing solution consists of a combination of compounds including methyl methacrylate at 25–50%, a photoinitiator, and SiO_2_ as a nanofiller. The presence of SiO2 facilitates interfacial crosslinking with the polymer matrix, thereby enhancing the physical and mechanical properties of the surface [33]. In a study conducted by Dos Santos et al. [34], SEM analysis was used to examine temporary prosthesis polymer surfaces after photopolymerization glazing. The findings showed that glazed samples exhibited less degradation and surface porosity compared to unglazed ones. Köroğlu A et al. [35] also noted that surface sealant agents significantly reduced surface roughness compared to conventionally polished specimens.

The color stability of provisional dental restoration materials is noticeably impacted by variables such as fluid uptake, degree of polymerization, surface texture, and material thickness [36]. In this current investigation, all the temporary resin samples were placed in a coffee solution for 15 days. The study’s findings revealed that immersing the temporary resin samples in a coffee solution for two weeks resulted in noticeable color changes. Various studies have revealed that polymer resins, especially when exposed to coffee, are more prone to discoloration than other substances such as tea, red wine, or curry solutions. Coffee contains tannin, a brown pigment known for staining teeth and dental restorative materials. Tannins are complex molecules with a medium-to-high molecular weight that can form insoluble compounds with carbohydrates and proteins. The low-polarity dyes in coffee effectively penetrate the polymer structure compared to high-polarity dyes [37]. Research has demonstrated that PMMA provisional resins are less likely to change color in comparison to BAMA. This outcome is consistent with the findings of a study conducted by Bayindir et al. [36], who also observed significant color changes in bis-acryl compared to methacrylate resins after immersion in coffee and red wine for 20–30 days at 37 °C. Furthermore, Kotnarin N et al. [38] found that PMMA resins were less prone to color change than BAMA resins, especially after longer immersion periods of up to 90 days. Elagra MI et al. [39] also found that bis-acryl resin composite materials showed significant color alteration, whereas PMMA materials exhibited superior color retention after immersing crowns in a tea solution for seven days. Some studies [16,40] have suggested that BAMA resin shows better color stability than PMMA acrylic resin. Differences in the results may be due to variations in materials, sample preparation, staining solution concentration, aging duration, and testing intervals.

The size distribution of filler particles, polarity of monomers, and pigment stability can result in varying degrees of polymerization, degree of cross-linking of resin molecules, water sorption, and consequently color stability [41]. The concentration of fillers can potentially correlate with their significant impact on solvent absorption and release within a polymer matrix. Dental polymer water absorption is affected by factors such as the density of the network, capacity for hydrogen bonding, and polar interactions [42]. The inclusion of monomers such as bisphenol A-diglycidyl dimethacrylate and triethylene glycol dimethacrylate, in combination with organic matrix and inorganic filler, serves to enhance flexural strength, decrease exothermic reaction, reduce polymerization shrinkage, and improve polishing capability [43]. The combination of these monomers within bis-acryl resins exhibits greater polarity compared to acrylic resin polymers. As a result, it increases the affinity of bis-acryl resin for polar liquid molecules, which leads to heightened absorption of staining substances [44]. Bis-acrylic resins’ heterogeneity also allows for increased penetration of pigment solutions into small material particles, leading to a higher degree of pigmentation [45]. PMMA-based resins with a more homogeneous composition are less prone to discoloration because they have a reduced ability to absorb and attract staining solutions [46].

The current research also assessed the influence of surface roughness on color retention. Findings suggested that negative control groups with higher Ra values were more prone to significant discoloration. The polished groups showed a reduced tendency for discoloration, whereas the application of surface glaze resulted in better resistance to color changes. The study’s findings reaffirm Koishi Y et al.’s [47] observation on the impact of specimen surface thickness and smoothness on color. The discoloration of samples results from both intrinsic and extrinsic processes. Intrinsic discoloration may be intensified by the penetration of pigments through microcracks or interfacial gaps at the filler–matrix contact over a rough surface. Extrinsic discoloration is also exacerbated by the adsorption of polar pigments and colorants present in the environment onto the rougher surface of resin composite materials [48]. A transparent layer of light-cured coating penetrates the surface of the resin, filling in micropores and defects. This effectively decreases porosity and reduces microleakage on the surface [49]. Dos Santos et al. [34] also noted that the use of glazing decreased the extent of color alteration caused by a coffee-based dye solution. Rutkunas et al. [15] and Almejrad et al. [49] similarly documented that glazing enhanced resistance to discoloration. Glazes applied to polymer surfaces can hinder water absorption and enhance surface lubricity, resulting in a hydrophobic surface. This modified surface exhibits lower protein adsorption compared to unglazed samples [50]. Previous studies [48,51] have indicated that a ΔE* ≥ 3.3 is deemed perceptible and clinically undesirable. In the current study, all experimental groups exhibited ΔE values exceeding 3.3, nevertheless, samples with surface glaze demonstrated color changes closer to what is considered clinically acceptable. Microbial biofilms on dental hard tissues in the oral cavity are the primary cause of tooth decay and discoloration of dental restorations. Key factors that influence bacterial adhesion include surface roughness, surface free energy, hydrophobicity of the restoration, and material composition. The rougher surface topographies offer protective microhabitats where microorganisms can evade mechanical cleaning forces such as brushing, muscle activity, and salivary flow. Consequently, inadequately finished restorations may facilitate the accumulation of biofilm on the surface and surrounding regions within the oral environment. Various polishing systems can be utilized to minimize this effect by eliminating surface irregularities and producing a smoother restoration surface.

The clinical implications of this study emphasize the importance of carefully selecting provisional restorative materials and following appropriate polishing protocols when fabricating provisional crowns. Proper material selection and polishing techniques are crucial for maintaining the esthetic appearance of provisional restorations and preserving patient satisfaction throughout the treatment process. When choosing the interim material and polishing methods, dentists must consider the individual patient’s risk factors for tooth discoloration, such as their dietary choices and oral hygiene practices. Limitations inherent in this in vitro study include replicating the complex oral environment, and factors such as fluctuations in pH, shifts in the oral microbiome, changes in temperature, masticatory forces, and behaviors like smoking could significantly impact the outcomes observed. Furthermore, the test samples utilized flat surfaces, whereas actual tooth surfaces present with cusps and grooves, posing a challenge to achieving consistent polishing in real clinical scenarios. Further research is necessary to substantiate the findings of this in vitro study through a long-term clinical investigation.

## 5. Conclusions

Within the limitations of this research, the findings indicated that unpolished acrylic bur-roughened provisional resin samples displayed higher Ra values. The tested polishing methods, including silicon carbide discs and diamond-coated rubber wheels, were effective at reducing the Ra values. However, a surface glaze application was able to achieve Ra values closer to the 0.2 μm threshold. In comparison to BAMA resin, PMMA resins exhibited superior color stability. Additionally, rougher surfaces tended to experience more noticeable color changes. The use of a surface sealant agent notably reduced material staining. The study findings emphasize the need to refine and polish provisional crowns prior to cementation in order to achieve superior color stability and surface morphology. Furthermore, future research is necessary to assess newer provisional materials and fabrication methods, including CAD-CAM and 3D printing technology, before their routine clinical use. Importantly, the study’s conclusions require corroboration through long-term clinical investigations.

## Figures and Tables

**Table 1:** Provisional crown materials used in the study.

Provisional Crown Resin	Composition	Manufacturer	Lot No	Shade
Polymethyl methacrylate (PMMA) resin	Powder: Polymethylmethacrylate (PMMA) and Benzoyl peroxide Liquid: Methylmethacrylate (MMA) and N, N-Dimethylp-toluidine	Trim^®^ Plus, Keystone Industries, Gibbstown NJ, USA	1403–130	A2
Methyl methacrylate-light-cure resin	Powder: poly(methyl methacrylate), dibenzoyl peroxide, iron(III) oxide, titanium dioxide, silicon dioxide. Liquid:methyl methacrylate (MMA), urethane dimethacrylate (UDMA)	UNIFAST™ LC. GC Corporation, Bunkyo-ku, Tokyo, Japan	1401.104	A2
Bisacryl methacrylate resin	Bis-Acryl Bisacryl methacrylate paste	Success CD, Promedica Dental Material GmbH, Neumuenster, Germany	161503	A2

**Table 2:** Mean (SD) surface roughness (Ra) values (µm) of different provisional resin groups.

Group	PMMA	BAMA	MMLC
Positive control	1.62(0.22)	1.41(0.18)	1.55(0.14)
Negative control	2.42(0.23)	2.59(0.20)	2.34(0.19)
DW	0.86(0.12)	1.10(0.27)	0.81(0.13)
CRT	0.36(0.01)	0.52(0.11)	0.28(0.05)
GA	0.17(0.07)	0.15(0.4)	0.24(0.04)

PMMA: Polymethyl methacrylate resin, BAMA: Bisacryl methacrylate resin, MMLC: Methyl methacrylate-light-cure resins, DW: Durawhite, CRT: Carepacy rubber polishing twists, GA: Glaze application.

**Table 3:** Mean ± SD color changes(ΔE) of the provisional resin samples from the baseline color and after surface conditioning and coffee immersion.

Group	PMMA	BAMA	MMLC
Positive control	5.66(0.31)	6.32(0.32)	5.28(0.47)
Negative control	6.59(0.57)	8.50(0.59)	6.31(0.48)
DW	4.90(0.55)	7.01(0.59)	5.12(0.54)
CRT	4.46(0.53)	5.87(0.44)	4.65(0.51)
GA	3.92(0.55)	5.39(0.63)	4.24(0.46)

**Table 4:** Tukey HSD post-hoc analysis of mean surface roughness between different polishing protocols.

Dependent Variable	I-Group	J-Group		
		PMMA	BAMA	MMLC
Surface roughness	PMMA	-	0.000 *	0.194
	BAMA	0.000 *	-	0.061
	MMLC	0.061	0.194	-

* Significant at the 0.01 level.

**Table 5:** Tukey HSD post-hoc analysis of mean color changes between different acrylic resin groups.

Dependent Variable	I-Group	J-Group		
		PMMA	BAMA	MMLC
Color changes	PMMA	-	0.000 *	1.000
	BAMA	0.000 *	-	0.000 *
	MMLC	1.000	0.000 *	-

* Significant at the 0.01 level.

## Data Availability

Available from corresponding author on reasonable request.

## References

[1] Guler A.U., Yilmaz F., Kulunk T., Guler E., Kurt S. (2005). Effects of different drinks on stainability of resin composite provisional restorative materials. J. Prosthet. Dent..

[2] Dede D.Ö., Şahin O., Koroglu A., Yilmaz B. (2016). Effect of sealant agents on the color stability and surface roughness of nanohybrid composite resins. J. Prosthet. Dent..

[3] Burns D.R., Beck D.A., Nelson S.K. (2003). A review of selected dental literature on contemporary provisional fixed prosthodontic treatment: Report of the Committee on Research in Fixed Prosthodontics of the Academy of Fixed Prosthodontics. J. Prosthet. Dent..

[4] Drago C. (2016). Frequency and type of prosthetic complications associated with interim, immediately loaded full-arch prostheses: A 2-year retrospective chart review. J. Prosthodont..

[5] Taşın S., Ismatullaev A., Usumez A. (2022). Comparison of surface roughness and color stainability of 3-dimensionally printed interim prosthodontic material with conventionally fabricated and CAD-CAM milled materials. J. Prosthet. Dent..

[6] Yao Q., Morton D., Eckert G.J., Lin W.S. (2021). The effect of surface treatments on the color stability of CAD-CAM interim fixed dental prostheses. J. Prosthet. Dent..

[7] Bitencourt S.B., Kanda R.Y., de Freitas Jorge C., Barão V.A., Sukotjo C., Wee A.G., Goiato M.C., Pesqueira A.A. (2020). Long-term stainability of interim prosthetic materials in acidic/staining solutions. J. Esthet. Restor. Dent..

[8] Patras M., Naka O., Doukoudakis S., Pissiotis A. (2012). Management of provisional restorations’ deficiencies: A literature review. J. Esthet. Restor. Dent..

[9] Maalhagh-Fard A., Wagner W.C., Pink F.E., Neme A.M. (2003). Evaluation of surface finish and polish of eight provisional restorative materials using acrylic bur and abrasive disk with and without pumice. Oper. Dent..

[10] Bennett H.E., Porteus J. (1961). Relation between surface roughness and specular reflectance at normal incidence. JOSA.

[11] Chung K.H. (1994). Effects of finishing and polishing procedures on the surface texture of resin composites. Dent. Mater..

[12] Ono M., Nikaido T., Ikeda M., Imai S., Hanada N., Tagami J., Matin K. (2007). Surface properties of resin composite materials relative to biofilm formation. Dent. Mater. J..

[13] Kaplan B.A., Goldstein G.R., Vijayaraghavan T.V., Nelson I.K. (1996). The effect of three polishing systems on the surface roughness of four hybrid composites: A profilometric and scanning electron microscopy study. J. Prosthet. Dent..

[14] Rizzante F.A., Bombonatti J.S., Vasconcelos L., Porto T.S., Teich S., Mondelli R.F. (2019). Influence of resin-coating agents on the roughness and color of composite resins. J. Prosthet. Dent..

[15] Rutkunas V., Sabaliauskas V., Mizutani H. (2010). Effects of different food colorants and polishing techniques on color stability of provisional prosthetic materials. Dent. Mater. J..

[16] Sham A.S.K., Chu F.C.S., Chai J., Chow T.W. (2004). Color stability of provisional prosthodontic materials. J. Prosthet. Dent..

[17] Strassler H.E., Anolik C., Frey C. (2007). High-strength, aesthetic provisional restorations using a bis-acryl composite. Dent. Today.

[18] Satou N., Khan A., Matsumae I., Satou J., Shintani H. (1989). In vitro color change of composite-based resins. Dent. Mater..

[19] Tupinambá Í.V., Giampá P.C., Rocha I.A., Lima E.M. (2018). Effect of different polishing methods on surface roughness of provisional prosthetic materials. J. Indian Prosthodont. Soc..

[20] Scheibe K.G.B.A., Almeida K.G.B., Medeiros I.S., Costa J.F., Alves C.M.C. (2009). Effect of different polishing systems on the surface roughness of microhybrid composites. J. Appl. Oral Sci..

[21] Faul F., Erdfelder E., Buchner A., Lang A.-G. (2009). Statistical power analyses using G* Power 3.1: Tests for correlation and regression analyses. Behav. Res. Methods.

[22] Paolone G., Mazzitelli C., Boggio F., Breschi L., Vichi A., Gherlone E., Cantatore G. (2023). Effect of Different Artificial Staining Procedures on the Color Stability and Translucency of a Nano-Hybrid Resin-Based Composite. Materials.

[23] Paul S., Peter A., Pietrobon N., Hämmerle C.H.F. (2002). Visual and spectrophotometric shade analysis of human teeth. J. Dent. Res..

[24] Vichi A., Louca C., Corciolani G., Ferrari M. (2011). Color related to ceramic and zirconia restorations: A review. Dent. Mater..

[25] Bollenl C.M., Lambrechts P., Quirynen M. (1997). Comparison of surface roughness of oral hard materials to the threshold surface roughness for bacterial plaque retention: A review of the literature. Dent. Mater..

[26] Erdemir U., Sancakli H.S., Yildiz E. (2012). The effect of one-step and multi-step polishing systems on the surface roughness and microhardness of novel resin composites. Eur. J. Dent..

[27] Gantz L., Fauxpoint G., Arntz Y., Pelletier H., Etienne O. (2021). In vitro comparison of the surface roughness of polymethyl methacrylate and bis-acrylic resins for interim restorations before and after polishing. J. Prosthet. Dent..

[28] Korkmaz Y., Ozel E., Attar N., Aksoy G. (2008). The influence of one-step polishing systems on the surface roughness and microhardness of nanocomposites. Oper. Dent..

[29] Neme A.L., Frazier K.B., Roeder L.B., Debner T.L. (2002). Effect of prophylactic polishing protocols on the surface roughness of esthetic restorative materials. Oper. Dent..

[30] Joiner A., Muller D., Elofsson U.M., Malmsten M., Arnebrant T. (2003). Adsorption from black tea and red wine onto in vitro salivary pellicles studied by ellipsometry. Eur. J. Oral Sci..

[31] Haselton D.R., Diaz-Arnold A.M., Dawson D.V. (2004). Effect of storage solution on surface roughness of provisional crown and fixed partial denture materials. J. Prosthodont. Implant. Esthet. Reconstr. Dent..

[32] Doray P.G., Eldiwany M.S., Powers J.M. (2003). Effect of resin surface sealers on improvement of stain resistance for a composite provisional material. J. Esthet. Restor. Dent..

[33] Gad M.M., Al-Harbi F.A., Akhtar S., Fouda S.M. (2022). 3D-printable denture base resin containing SiO_2_ nanoparticles: An in vitro analysis of mechanical and surface properties. J. Prosthodont..

[34] Dos Santos D.M., Commar B.C., da Rocha Bonatto L., da Silva E.V., Sônego M.V., Rangel E.C., Pesqueira A.A., Goiato M.C. (2017). Surface characterization of polymers used in fabrication of interim prostheses after treatment with photopolymerized glaze. Mater. Sci. Eng. C.

[35] Köroğlu A., Sahin O., Dede D.Ö., Yilmaz B. (2016). Effect of different surface treatment methods on the surface roughness and color stability of interim prosthodontic materials. J. Prosthet. Dent..

[36] Bayindir F., Kürklü D., Yanikoğlu N.D. (2012). The effect of staining solutions on the color stability of provisional prosthodontic materials. J. Dent..

[37] Bravo L. (1998). Polyphenols: Chemistry, dietary sources, metabolism, and nutritional significance. Nutr. Rev..

[38] Kotnarin N., Nagaviroj N., Kanchanavasita W. (2018). The effect of staining solutions on the color stability of the provisional restorative materials. Mahidol Dent. J..

[39] Elagra M.I., Rayyan M.R., Alhomaidhi M.M., Alanaziy A.A., Alnefaie M.O. (2017). Color stability and marginal integrity of interim crowns: An in vitro study. Eur. J. Dent..

[40] Doray P.G., Li D., Powers J.M. (2001). Color stability of provisional restorative materials after accelerated aging. J. Prosthodont..

[41] Jalali H., Dorriz H., Hoseinkhezri F., Razavi S.E. (2012). In vitro color stability of provisional restorative materials. Indian J. Dent. Res..

[42] Al-Akhali A.A.-R.M., Al-Hamzi M., Al-Shami I.Z., Al-Kholani A.I., Madfa A.A. (2023). Effect of khat extract on color stability of digitally and manually fabricated provisional restorations: An in vitro comparative study. BMC Oral Health.

[43] Haselton D.R., Diaz-Arnold A.M., Dawson D.V. (2005). Color stability of provisional crown and fixed partial denture resins. J. Prosthet. Dent..

[44] Mickeviciute E., Ivanauskiene E., Noreikiene V. (2016). In vitro color and roughness stability of different temporary restorative materials. Stomatologija.

[45] Turgut S., Bagis B., Ayaz E.A., Ulusoy K.U., Altintas S.H., Korkmaz F.M., Bagis N. (2013). Discoloration of provisional restorations after oral rinses. Int. J. Med. Sci..

[46] Gujjari A., Bhatnagar V., Basavaraju R. (2013). Color stability and flexural strength of poly (methyl methacrylate) and bis-acrylic composite based provisional crown and bridge auto-polymerizing resins exposed to beverages and food dye: An: In vitro: Study. Indian, J. Dent. Res..

[47] Koishi Y., Tanoue N., Matsumura H., Atsuta M. (2001). Colour reproducibility of a photo-activated prosthetic composite with different thicknesses. J. Oral Rehabil..

[48] Um C.M., Ruyter I. (1991). Staining of resin-based veneering materials with coffee and tea. Quintessence Int..

[49] Almejrad L., Yang C.C., Morton D., Lin W.S. (2022). The effects of beverages and surface treatments on the color stability of 3D-printed interim restorations. J. Prosthodont..

[50] Rechendorff K., Hovgaard M.B., Foss M., Zhdanov V.P., Besenbacher F. (2006). Enhancement of protein adsorption induced by surface roughness. Langmuir.

[51] Eldiwany M., Friedl K.H., Powers J.M. (1995). Color stability of light-cured and post-cured composites. Am. J. Dent..

